# Prognostic impact of the expression of Wnt-signaling proteins in cervical carcinoma FIGO stage I-IV treated with radiotherapy or chemoradiotherapy

**DOI:** 10.18632/oncotarget.11642

**Published:** 2016-08-26

**Authors:** Louise Bohr Mordhorst, Cecilia Ahlin, Bengt Sorbe

**Affiliations:** ^1^ Department of Oncology, Örebro University Hospital, Örebro, Sweden

**Keywords:** cervical carcinoma, Wnt signaling pathway, β-catenin, APC

## Abstract

Wnt signaling proteins were assessed in patients with primary cervical carcinomas who received chemoradiation. The associations between three Wnt signaling proteins and prognosis were assessed. Specimens from 122 patients with cervical carcinomas (FIGO stage I-IV) were immunohistochemically (IHC) analyzed for β-catenin, APC and axin protein expression. Associations between these Wnt-protein expressions, clinicopathological factors, and clinical outcome data were examined.

Positive IHC staining for the β-catenin protein (cell-membranes, cytoplasm and nuclei) was recorded in 88%, 58% and 5%, respectively. There was a strong association between β-catenin staining of the cell-membranes and prediction of recurrences and prognosis (*p* = 0. 002). Tumors with > 5% of nuclear β-catenin staining were associated with inferior cancer-specific survival (*p* = 0.048) compared with no staining. The overall recurrence rate was significantly higher in the group with increased nuclear staining (67%) compared with the group with no staining (33%). Nuclear APC staining of high intensity was associated with a significantly worse cancer-specific survival and increased overall recurrence rate compared to tumors with weak staining. Distant recurrences were recorded in 29% of cases with intense staining and in 14% of cases with low staining.

The Wnt signaling pathway seems to be of importance in the process of cervical oncogenesis. A predictive and prognostic value was found for β-catenin, where strong cell-membrane staining was favorable, and > 5% positive nuclear staining was associated with poorer cancer-specific survival and overall recurrence rate. Nuclear APC staining intensity was also associated with a less favorable prognosis.

## INTRODUCTION

Cervical carcinoma is the fourth most common cancer in women, and the seventh overall, with estimated 528,000 new cases and 266,000 deaths from this viral-induced cancer worldwide in 2012 [1].

In Sweden, 467 new cases were recorded in 2013, accounting for 1.8% of all female cancers, and 142 deaths. The incidence of cervical carcinoma has declined in most European countries due to well organized screening programs. The relative 5-year survival rate in Sweden 2013 was 68% (95% confidence interval 67-70%) [2]. In the developed world most patients present with early disease, either confined to the cervix or with limited extension beyond it (FIGO stages IB1-IIA). Radical hysterectomy with node dissection and radical radiotherapy are two options both giving 5-year survival rates of 80-90%. For locally advanced disease (FIGO stages IIB-IVA) as well as bulky stage IB (> 4 cm) radical radiotherapy has successfully been “state of the art” for more than a century [3, 4]. External pelvic radiotherapy (EBRT) gives a good chance of cure, but the radiation dose is limited by normal tissue tolerance. Intracavitary brachytherapy (ICBT) plays an important role in the management of patients with cervical cancer in most stages (I-IV). The steep dose gradient allows a high central dose to the tumor, while sparing the risk organs [5]. The combination of external beam radiotherapy (EBRT) and intracavitary brachytherapy (ICBT) [6] and concomitant chemotherapy has become standard of care for locally advanced disease [7].

The 5-year survival rate varies from 60% for patients with stage IIB disease to 20% for patients with stage IVA disease, and for recurrent cervical cancer it ranges from 10 to 22% [7]. New targets and combined treatment strategies are therefore urgently needed.

The Wnt/β-catenin signaling pathway is a highly conserved, intracellular signaling mechanism which plays an essential role for numerous key cellular processes. Three Wnt signaling pathways have been characterized: (1) The canonical Wnt pathway, (2) the noncanonical planar cell polarity pathway, and (3) the noncanonical Wnt/calcium pathway. All three pathways are activated by binding a Wnt-protein ligand to a Frizzled family receptor together with a co-receptor (LRP5/6), which passes the biological signal inside the cell. The canonical Wnt pathway, the one most well understood, leads to regulation of gene transcription. The noncanonical planar cell polarity pathway regulates the cytoskeleton that is responsible for the shape of the cell. The noncanonical Wnt/calcium pathway regulates calcium inside the cell.

Wnt signaling was first identified for its role in carcinogenesis and, then for its function in embryonic development. Wnt signaling also controls tissue regeneration in adult bone marrow, skin and intestine. Later research found that the genes responsible for these abnormalities also influenced breast cancer development in mice. Consistent alterations of some important pathways controlling cell proliferation and apoptosis, such as Wnt/β-catenin, has been identified in different types of cancer, like hepatocellular cancer, colorectal cancer, gastric cancer, osteosarcoma, and breast cancer. Later, this signaling pathway was also implicated in oropharyngeal, and cervical cancer.

Persistent infection with Human Papilloma Virus classified as high-risk HPV (hr-HPV), is a necessary factor for development of cervical carcinomas and their viral oncogenes interact and regulate the function of several cellular proteins. A participation of the Wnt/β-catenin signaling pathway in HPV-related cancers and the possible mechanisms by which HPV E6 and E7 oncoproteins induce the activation of this pathway has been reported [8].

The multifunctional protein β-catenin was initially described as a cell-cell adhesion molecule as a link between cadherin and the actin cytoskeleton [9]. Aberrant functions may result in increased cell motility, invasion and metastasis in several epithelial carcinomas [10, 11]. β-catenin is also a critical downstream mediator of the Wnt signaling pathway during embryogenesis and tumorigenesis [12–14]. Under normal conditions, β-catenin is located sub membranous. The cytoplasmic β-catenin is regulated by the adenomatous polyposis coli (APC) tumor suppressor protein, axin and the glycogen synthase kinase-3β (GSK-3β) complex, which phosphorylates β-catenin resulting in rapid ubiquitin-proteasome degradation [15]. Mutations in APC, axin or β-catenin itself, as well as the activation of the Wnt signaling pathway, have been demonstrated to promote the accumulation of β-catenin in the cytoplasm with a subsequent translocation of the protein into the nucleus. In the nucleus, β-catenin functions as an oncogene influencing cell cycle control and cellular proliferation [16–19].

Changed β-catenin expression has been shown in a variety of human cancer types [20–25], and a number of studies have reported the clinicopathological significance of the Wnt signaling pathway in human cervical carcinomas.

However, data regarding its predictive and prognostic value and its role in tumor progression in cervical carcinoma is limited. Thus, in the present study we have studied the expression of β-catenin, axin and APC in a consecutive series of 122 cervical cancer (FIGO stages I-IV) patients treated with external beam radiotherapy and brachytherapy (with or without concomitant chemotherapy) by immunohistochemistry (IHC) using tissue microarray (TMA). The ratio of expression, intensity and location of the three proteins were examined to evaluate and assess a possible relationship with clinical and pathological predictive and prognostic factors and treatment efficacy.

## RESULTS

The clinical and pathological characteristics of the complete series are presented in Table 1.

**Table 1:** The clinical and pathological characteristics of the complete series (n = 122)

Characteristics	No. of patients (%)
Median age (range) years	68 (30-90)
**FIGO stage**	
IB	27 (22)
IIA	31 (25)
IIB	41 (34)
IIIA	4 (3)
IIIB	14 (11)
IVA	2 (2)
IVB	3 (2)
**Grade**	
Well differentiated	8 (7)
Moderately well differentiated	56 (46)
Poorly differentiated	58 (48)
**Type of histology**	
Adenocarcinoma	19 (16)
Adenosquamous carcinoma	3 (2)
Squamous cell carcinomas	100 (82)
**Tumor size**	
Diameter (median, range) mm	40 (15-90)
**DNA ploidy**	
Diploid	44 (36)
Aneuploid	62 (51)
Tetraploid	8 (7)
Unknown	8 (7)
**HPV status**	
HPV16	54 (44)
HPV18	12 (10)
HPVother	31 (25)
Unknown	25 (21)

Other HPV types: 31, 33, 35, 39, 42, 45, 51, 52, 53, 56, 58, 59, 68, 70, 73

### β-catenin

The membranes of the tumor cells stained positively in 87.9% (95% CI: 83.6-92.3%) in mean of the analyzed sections. The corresponding positive staining in the cytoplasm was 58.5% (95% CI 51.1-65.9%) and in the nuclei 5.4% (95% CI 2.6-8.2%) (Table 2). The intensity distribution of the membrane staining was low in 35.1%, medium in 51.6%, and high in 13.3%. In 48 out of 121 tumors (39.7%) the membranes were intensely stained in one or more of the evaluated cells (Table 3). The mean H-score for the IHC staining of the tumor cell membranes was 178.2 (95% CI 169.1-187.3) and the range was 100-300. The H-score was a weak and mostly non-significant predictive and prognostic factor in this series. A cut-off level of 0 (= 0 *vs*. > 0) for β-catenin staining was not a relevant definition of negative and positive cases in this study and this was true for all staining sites analyzed (membrane, cytoplasm, and nucleus). On the other hand, and the presence of any intense staining of the lateral membranes (Figure 2) and a nuclear staining of 5% or more (Figure 3), were of significant predictive and prognostic importance. No predictive or prognostic impact, irrespective of cut-off levels, was found regarding the percentage or intensity of cytoplasmic staining.

**Figure 1:**
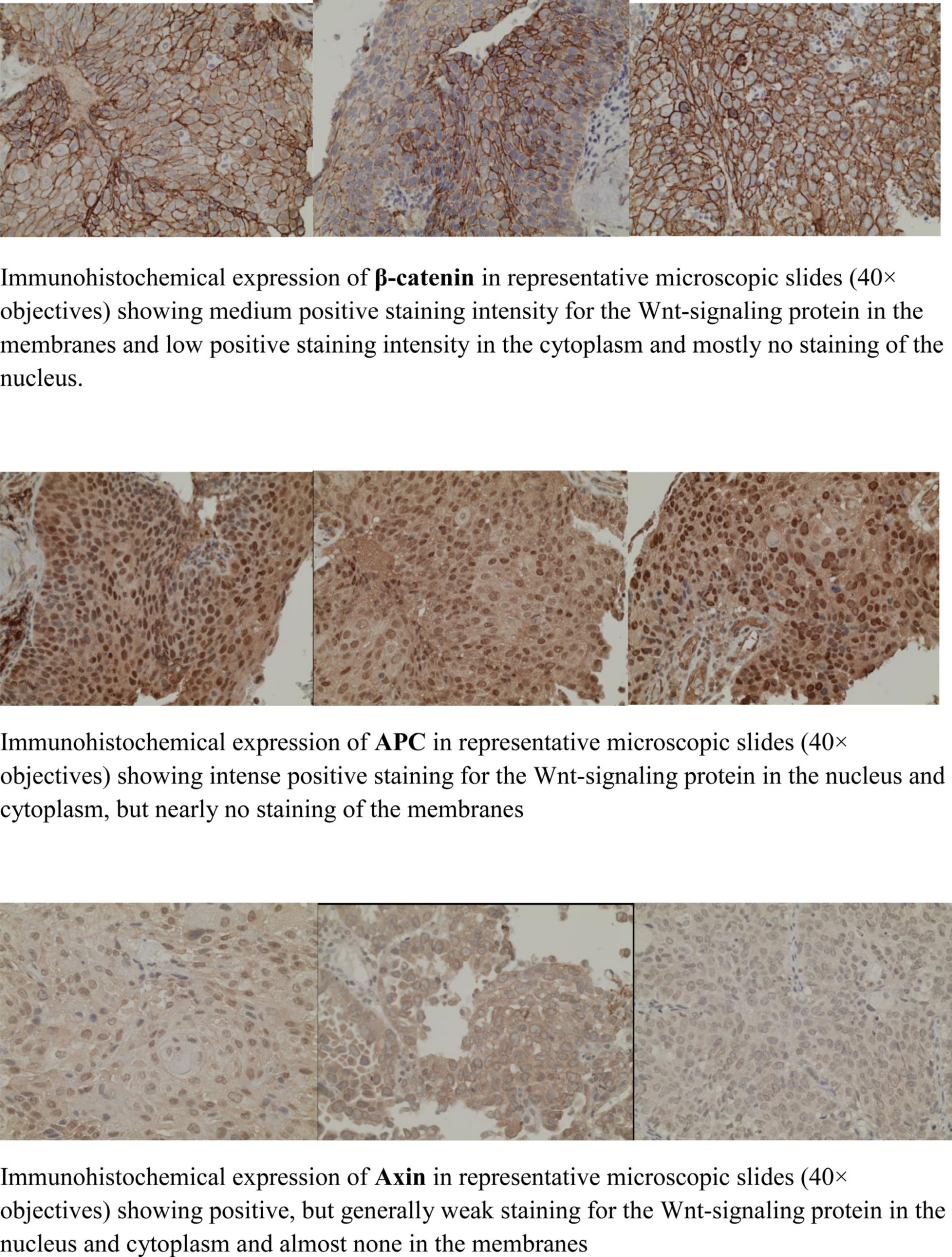
Microscopic slides of immunohistochemical staining of the Wnt-signaling proteins β-catenin, PC and axin Illustrating the data in Table 2 and 3.

**Figure 2:**
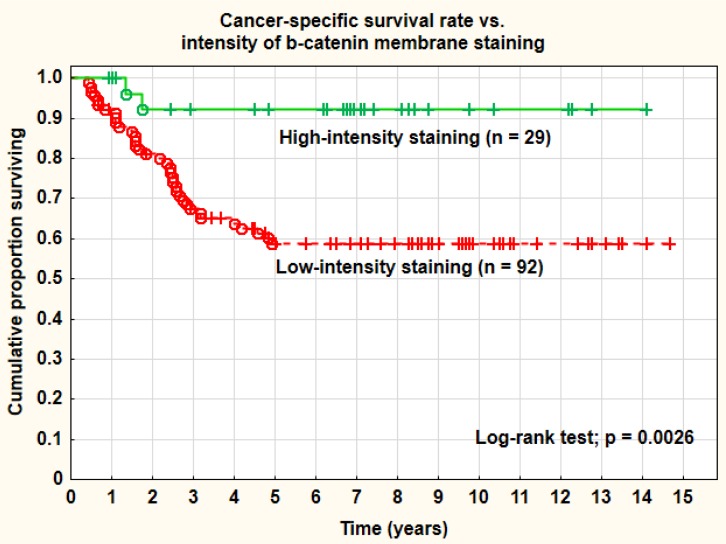
Cancer-specific survival rate *vs*. **intensity of β-catenin membrane staining**

**Figure 3:**
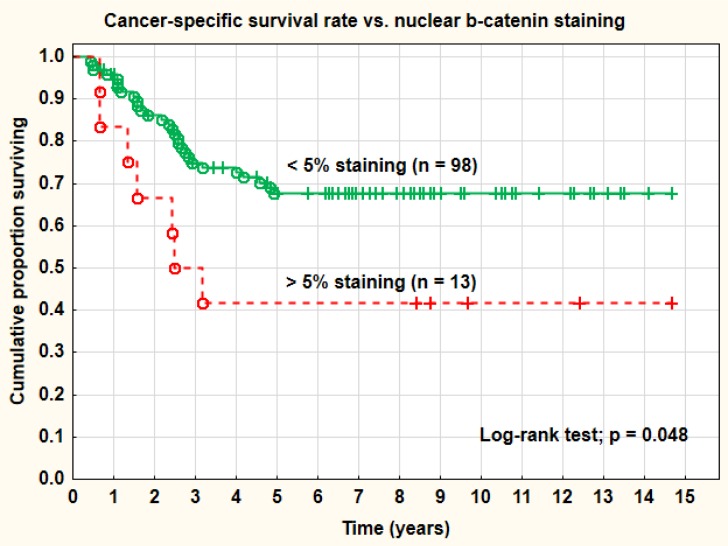
Cancer-specific survival rate *vs*. nuclear β-catenin staining

**Table 2:** Immunohistochemical staining (IHC) of β-catenin, axin and APC in the membranes, cytoplasm and nucleus of cervical cancer cells

Protein	No.	Mean	[95% CI]	Range
**β-catenin**	121			
Nucleus		5.4%	[2.6-8.2%]	0-80%
Cytoplasm		58.5%	[51.1-65.9%]	0-100%
Membrane		87.9%	[83.6-92.3%]	0-100%
**Axin**	118			
Nucleus		41.2%	[34.6-47.8%]	0-100%
Cytoplasm		88.8%	[84.1-93.6%]	0-100%
Membrane		1.9%	[0.1-3.8%]	0-100%
**APC**	122			
Nucleus		89.2%	[85.2-93.2%]	0-100%
Cytoplasm		94.3%	[90.4-98.1%]	0-100%
Membrane		2.4%	[0.2-4.9%]	0-95%

Percentages of positive cells.

**Table 3:** Intensity of immunohistochemical staining (IHC) of β-catenin, axin and APC in the membranes, cytoplasm and nucleus of cervical cancer cells

Protein	No.	IHC staining intensity		
**β-catenin**	121	**Low**	**Medium**	**Strong**
Nucleus		93.5%	6.3%	0.2%
Cytoplasm		76.8%	20.5%	2.6%
Membrane		35.1%	51.6%	13.3%
**Axin**	118			
Nucleus		83.5%	14.3%	2.2%
Cytoplasm		85.9%	13.4%	0.7%
Membrane		57.1%	28.6%	14.3%
**APC**	122			
Nucleus		41.2%	51.6%	7.2%
Cytoplasm		68.8%	29.3%	1.9%
Membrane		100.0%	0.0%	0.0%

Percentage distributions.

Increased percentage (> 5%) of β-catenin nuclear staining was associated with a worse prognosis and a significantly (log-rank test; *p* = 0.048) inferior cancer-specific survival rate compared with no nuclear staining (41.7% *vs*. 67.8% at 5 years). Adenocarcinomas showed a higher percentage (33.3%) of more than 5% nuclear staining than squamous cell carcinomas (14.3%), but a lower percentage (23.8% *vs*. 43.0%) of intense membrane staining. These differences did not reach statistical significance, however (Pearson chi-square test; *p* = 0.092 and 0.102). The staining intensity of the cell membranes (strong *vs*. weaker staining intensity) was also a highly significant (log-rank test; *p* = 0.0026) prognostic factor for cancer-specific survival rate. The intensity of cell membrane staining was also statistically significant (*p* < 0.05) after correction for tumor stage, tumor size, and histology in a Cox multivariate proportional regression analysis. Strong staining intensity of the cell membranes was a favorable prognostic factor. The overall recurrence rate was significantly (Pearson chi-square test; *p* = 0.021) higher in the group with increased nuclear staining (66.7%) compared with the group with no staining (32.7%). The overall recurrence rate was also higher in tumors with weak membrane staining (41.3%) than in tumors with strong membrane staining (10.3%). This difference was statistically highly significant (Pearson chi-square test; *p* = 0.0021). Differences in distant recurrences, analyzed separately, were not statistically significant. The primary cure rate (complete remission) of the tumors was not associated with the intensity of the membrane staining. There was a tendency to lower intensity of membrane staining in tumors in more advanced clinical stages (FIGO III-IV), more pronounced in adenocarcinomas, but not statistically significant. In a study by Imura et al. on 51 adenocarcinomas, treated by radical surgery, expression of β-catenin was highly significantly associated with surgical tumor stage. There were no significant associations between types of HPV (HPV16 *vs*. other types or HPV18 *vs*. other types) and the significant β-catenin tumor markers.

### β-catenin staining and concomitant chemotherapy

Tumors with strong membrane staining must be regarded as “low-risk” cases and with a more favorable prognosis. The use of concomitant chemotherapy in this group improved the 5-year cancer-specific survival rate from 75.3% to 83.3% (8.0%) which was a non-significant change (log-rank test; *p* = 0.505). On the other hand, in the group with weak β-catenin staining of the membranes, regarded as a “high-risk” group, the corresponding improvement in survival was from 49.7% to 71.0% (21.3%) at 5 years (log-rank test; *p* = 0.029). Thus, this membrane staining pattern might have an impact on treatment planning defining a high-risk group that benefit from concomitant chemotherapy.

### APC

**Figure 4:**
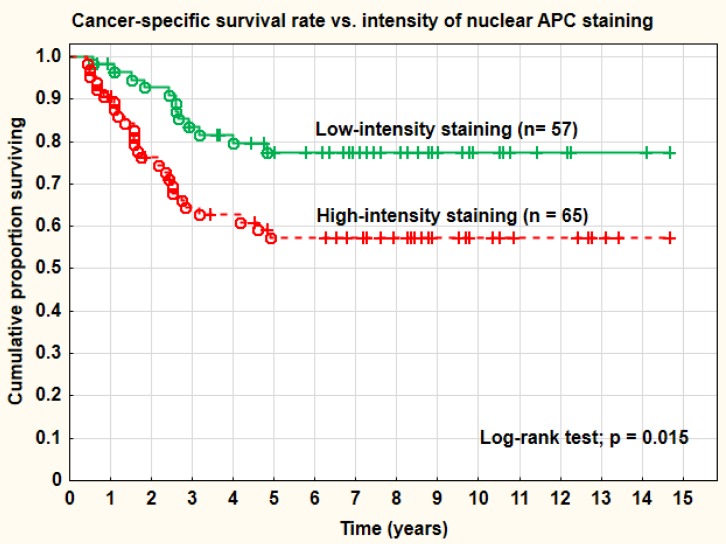
Cancer-specific survival rate *vs*. intensity of nuclear APC staining

A cut-off level of 0 (= 0 *vs*. > 0) for APC staining was not a relevant definition of negative and positive cases as for β-catenin in this study and this was true for all staining sites analyzed (membrane, cytoplasm, and nucleus). Intense nuclear APC staining was noted in 65 tumors (53.3%), and this staining pattern was associated with a significantly (log-rank test; *p* = 0.015) worse cancer-specific survival rate (57% at 5 years) compared with tumors with only mild or medium staining intensity (68% at 5 years) (Figure 4). The overall recurrence rate (43.1% *vs*. 22.8%) was also significantly (Pearson chi-square test; *p* = 0.018) associated with the intensity of the nuclear staining. Distant recurrences were recorded in 29.2% of cases with intense nuclear APC-staining and in 14.0% of cases with low-medium staining (Pearson chi-square test; *p* = 0.044). Intense nuclear APC-staining was similar for squamous carcinomas and adenocarcinomas. However, in the cytoplasm the intensity of the staining was of no predictive or prognostic importance. Overall, 118 out of 122 tumors (96.7%) stained positively for APC in the cytoplasm. The lateral membranes were in most cases not stained by APC. Data of hypermethylation of the APC1-gene from a prior study was not significantly (Pearson chi-square test; *p* = 0.403) associated with intense nuclear APC-staining in this study, despite a significant association with distant metastases for both.

### Axin

Nuclear staining was recorded in 41.2% (95% CI 34.6-47.8%) in mean of the evaluated tumor cells. On the other hand, the mean staining of the cytoplasm was 88.8% (95% CI 84.1-93.6%) (Table 2). The mean H-score for the cytoplasmic staining was 110.1 (95% CI 103.4-116.7). The H-score of axin was of no predictive or prognostic value. The membranes of most tumor cells stained negatively or with low intensity. The staining of axin (percentage or intensity) in the nucleus or in the cytoplasm was not of significant predictive or prognostic value in this series of cervical carcinomas.

### Correlations

There was a significant (*p* < 0.05) positive correlation (r = 0.270) of cytoplasmic staining between APC and axin, but not for β-catenin. No significant correlations were noted for nuclear and membrane staining of the three proteins. However, intense nuclear APC staining was noted in 72.7% of the cases with > 5% β-catenin staining and in 50.0% of the cases with no nuclear β-catenin staining (Pearson chi-square; *p* = 0.053).

## DISCUSSION

Cervical carcinoma is a highly HPV-related disease. A persistent infection with one or several high-risk HPV strains is a necessary prerequisite for cancer development [28]. However, this infection alone is not enough in the process of carcinogenesis, but other changes in one or more signal pathways are required for a malignant transformation of normal epithelial cells [8]. In our series 80% (97/122) of the tumors were analyzed and found to be HPV-positive and of these 68% were positive for HPV16 (54/97) or HPV18 (12/97).

The Wnt/β-catenin pathway is a pathway among others that may be involved together with HPV-proteins (E6 and E7) in the malignant changes of the cervical epithelial cells. Not only carcinogenesis per se but also cell proliferation, differentiation, cell-cell adhesion [29], and cell death (apoptosis) in established malignancies are controlled via biochemical signals in these pathways. Our series of cervical carcinomas were treated with combined radiotherapy, external pelvic beam therapy and brachytherapy, and as we know cell apoptosis is an important mechanism for the treatment effect and death of the tumor cells after radiotherapy. Cell proliferation and apoptosis are regulated by a number of pathways, among them activation of the wnt/β-catenin pathway, and this has been found in osteosarcoma [30], hepatocellular carcinoma [31], colorectal carcinoma [32], breast carcinoma [33], and recently also in oropharyngeal [34] and cervical carcinomas [35]. The Wnt canonical signaling pathway is the best understood of at least three signaling pathways activated by the Wnt ligands. In the absence of the Wnt ligands, β-catenin is mainly located at cellular junctions (membranes), but a small amount is also found in the cytoplasm and binds to a complex responsible for degradation of β-catenin by an ubiquitin/proteasome. This complex consists of the protein axin (a scaffold protein) and among others also APC and GSK3β recruited by axin. Wnt ligand binding to the Frizzled receptor delocalizes β-catenin from the membranes, with accumulation in the cytoplasm and the nucleus. The HPV proteins E6 and E7 are shown to be involved in β-catenin nuclear accumulation [34]. In the nucleus, β-catenin binds to transcriptional factors and interactions with β-catenin in the nucleus promote expression of diverse genes regulating cellular polarity, proliferation and differentiation, such as c-jun, c-myc, cyclin D1, axin-2, and Tcf-1.

In the present study, we therefore decided to study β-catenin itself, but also axin and APC and their localization in the cell membranes, cytoplasm and or the nucleus. The aim of the study was to find out if the immunohistochemical staining of these proteins in the tumor cells could be used as predictive and prognostic tumor markers in cervical carcinomas, mainly in advanced clinical stages, treated with primary radiotherapy. Much effort was devoted to evaluation of the immunohistochemistry. Percentage of stained cells as well as the intensity of staining was evaluated as well as the site of the staining (membrane, cytoplasm or nucleus). At least 200 tumor cells were individually analyzed per case. For β-catenin the site of expression is important since it is associated with the function of the protein. High expression of staining and intensity of the membranes signals something quite different than high nuclear staining. During tumor progression there is a shift in localization from the plasma membranes to the cytoplasm and the nucleus. In many studies using immunohistochemistry, not only for the wnt/ β-catenin pathway, not enough attention has been paid to this important fact [38].

With regard to predictive and prognostic impact β-catenin seemed to be the most important one of the three analyzed proteins in this study. The β-catenin protein was frequently observed at the membranes in 88% of the carcinomas. Other authors have reported a frequency of 75% in the membranes [35]. A typical high staining intensity of the membranes and low or absent staining of the cytoplasm and nucleus was associated with favorable clinical and histopathological tumor characteristics and treatment outcome of the disease. This is also the distribution seen in normal tissue [33, 35]. Thus, intensity of the membrane staining was significantly associated with the overall recurrence rate and cancer-specific survival rate. However, the primary cure rate (complete remission) of the tumors after completed radiotherapy (achieved in 92% in this series) was not associated with membrane staining. The E-cadherin/catenin adhesion complex is of importance for cervical cancer invasion and the expression is reduced compared with normal cervical tissue and carcinoma in situ [29].

Nuclear staining was surprisingly low in our study with only 5% positive staining in mean of the nuclei. This finding was in agreement with the report by Rodrigeuz-Sastre et al. [35]. Despite the low nuclear staining percentage it was found that more than 5% nuclear staining was associated with increased recurrence rate and a significantly worse cancer-specific survival rate. In contrast to the situation in cervix cancer as many as 63% of breast cancers are positive for β-catenin in the nucleus [37] and 19% of colorectal carcinomas [38]. Adenocarcinoma, a known unfavorable prognostic histology in cervix cancer, was also more frequent in this group of tumors with increased nuclear β-catenin staining.

Staining of the cytoplasm was not found to be a predictive or prognostic factor in this series. This is in contrast to the situation in basal-like breast cancer where cytosolic β-catenin was significantly and independently associated with a worse overall survival rate [31].

An interesting finding in this series was the association between week membrane staining of β-catenin, indicating a high-risk tumor, and improved treatment effect of concomitant chemotherapy with weekly cisplatin. The cancer-specific survival rate was improved with more than 20% in this risk group with the addition of cisplatin compared to only 8% in a low-risk group with high-intensity staining of the membranes. There is no report of this finding in the literature before. A potential use of this marker for treatment planning is an option in the future.

Regarding APC an intense nuclear staining was noted in 53% of the cases and this staining pattern was a significant predictive factor for tumor recurrences, both overall and at distant sites. High intensity nuclear staining was also a prognostic factor associated with a significantly worse cancer-specific survival rate. Type of histology was not associated with the APC-staining intensity. Staining of the cytoplasm was frequent with 97% positive staining, but the staining intensity was not of predictive or prognostic value. Less than 3% of the cells showed any membrane staining for APC. An interesting finding was higher frequency of intense nuclear APC-staining in tumors with increased (> 5%) β-catenin nuclear expression. This finding indicates an interaction of the two proteins in the nucleus that might regulate the nuclear β-catenin effect.

The axin protein, despite part of the complex in the cytoplasm, regulating the β-catenin activity and transfer to the nucleus, was not alone a significant predictive or prognostic factor in this series of cervical carcinomas. However, there was a significant correlation between axin and APC staining in the cytoplasm, probably depending on their interaction in the β-catenin regulating complex.

The importance of a careful immunohistochemistry technique and detailed evaluation of the staining pattern for the various signal proteins was obvious in this study. The evaluation must be done with functional knowledge of the pathway and the individual proteins in mind. Both frequency and staining intensity must be taken into account as well as the site of staining in the tumor cell. Staining of β-catenin was a good example of this, but this way of working should probably be a general policy in evaluation of all immunohistochemical studies.

The Wnt/ β-catenin pathway seems to be an important pathway in many cancers during carcinogenesis and not at least for HPV-dependent carcinomas, like cervical cancer. In our study we have shown its importance also in established, invasive carcinomas. β-catenin signaling is of importance in sustaining the phenotype of cancer stem cells and to maintain epithelial tumors [39]. Radiotherapy, not so much studied in this context before, was chosen for more advanced tumors, not suitable for surgery, and the treatment effect of irradiation of tumors is dependent on apoptosis. The intensity of membrane staining and accumulation of β-catenin in the tumor cell nucleus was of both predictive and prognostic impact for tumor recurrences, response to concomitant chemotherapy and cancer-specific survival rate. However, in a series of 219 squamous cell carcinomas treated with radical surgery in Norway, E-cadherin, alpha-, beta-, and gamma-catenin were not independently associated with prognosis in stage IB [40]. Thus, type of therapy and tumor stage must be taken into account when predictive and prognostic factors are evaluated in cervical cancer. The APC protein, known to be part of the regulatory complex of β-catenin in the cytoplasm, was also associated with β-catenin in the cell nucleus, probably as a potential nuclear exporter of β-catenin protein [41].

Since an aberrant Wnt pathway is present in many cancers therapeutic intervention in tumors containing defects in this pathway is a possibility for the future. Inhibitors of enzymes that regulate the stability of the β-catenin destruction complex, and tailored peptides that disrupt the interaction of β-catenin with its transcriptional partners TCF4 and BCL9 in the nucleus are possible points for actions of new pharmaceutical agents and thus possible improvement in cancer treatment [32, 42].

## MATERIALS AND METHODS

### Patients

In all, 122 patients with invasive cervical carcinomas were included in the series (Table 1). All patients were treated with a combination of external beam pelvic irradiation (EBRT) and brachytherapy. A 4-field box technique was used to treat the pelvis with an external beam dose of 50 Gy in 2.0 Gy daily fractions, five times a week, delivered using 18 megavolt photons. The intracavitary brachytherapy (ICBT) was given during the external treatment as 5 intrauterine treatments of 6.0 Gy each, using high-dose-rate (Ir-192) equipment. In 44 patients (36%) concomitant chemotherapy (weekly cisplatin, 40 mg/m^2^) was administered. The mean age of the patients was 65 years (range 30-90 years). Tumor characteristics are presented in Table 1. Lymph node dissection was not performed at the FIGO-staging of the tumors, and lymph node size at CT-image was not taken into account in treatment planning. Tumor samples were collected between 1993 and 2007. All tissue samples were collected before any type of therapy was instituted. The study was approved by the Ethics Committee in Uppsala, Sweden.

### Clinical samples

A consecutive series of tumor specimens were identified and a total of 122 cases of formalin-fixed and paraffin-embedded tumor blocks from punch biopsies were obtained from the department of Pathology, Örebro University Hospital. Tissue microarray slides were employed for the purpose of effective analyses. For preparation of these slides, we punched two tissue columns from the original blocks and inserted them into new paraffin blocks (Tissue microarray Pathology devices, USA) each containing 4 × 24 holes to accept the tissue columns. Then, serially sectioned slides were prepared. Each tissue microarray slide could hold 96 specimens, allowing us to analyze 96 specimens simultaneously with a minimum of variation during the staining process. Each specimen was round in shape and 1.0 mm in diameter, thereby providing a sufficient amount of tissue for histopathological analysis. In 118 to 122 cases it was technically possible to evaluate the immunohistochemical staining of all three proteins. All 122 samples were analyzed for at least one or more of the three proteins.

### Immunohistochemistry

Sections (4 μM) were prepared on superfrost microscope slides (Menzel gläser, Germany). The sections were deparaffinized in xylene twice for 5 minutes, rehydrated in a descending series of ethanol (99%, 96%, and 70%), and followed by washes in distilled water. Antigen retrieval was achieved by heating the samples in retrieval buffer with low pH 6.2 (DIVA Decloacer Biocare Medical, USA) or high pH 9.5 (BORG Decloacer Biocare Medical, USA) using Decloaking chamber tm NxGen (Biocare Medical, USA) at 110°C for 15 minutes. Then the sections were washed in wash buffer (TBS Auto wash buffer, Biocare Medical, USA). Staining was performed using MACH1 Universal HRP polymer detection kit and Immunostainer Intellipath (Biocare Medical, USA) according to the manufacturer's protocol. The slides were incubated 30 minutes with three primary antibodies (anti-axin, anti-APC and anti-β-catenin). The antibodies and retrieval buffers used are presented in Table 2. The slides were counterstained with Mayer's Hematoxyline (Histolab Products, Sweden), and mounted with PERTEX for examination.

### Evaluation of the immunohistochemistry

The percentage of positive stained tumor cells in the membrane, cytoplasm and nucleus and the intensity (weak, moderate and strong) of the staining of the cell-membrane, cytoplasm and nucleus were calculated for every specimen examined (Figure 1). In all, more than 200 tumor cells were evaluated and registered for 12 parameters per cell per patient specimen (Tables 3).

β-catenin staining was expressed mainly in the membrane and the cytoplasm. APC in both cytoplasm and nucleus, and axin staining was expressed mainly in the cytoplasm. Each specimen was examined in high-power field microscope (40 x objectives) and scored by one of the authors (LBM), two times, blinded. A limited number (10 tumors) was examined by two of the investigators (LBM, CA) independently of each other. There was a good agreement between the two investigators regarding percentage and intensity of staining.

Mean and median values were calculated as well as the 95% confidence interval. The median value was used as cut-off point in some analyses to define predictive and prognostic groups with up- and down-regulation of the three proteins. A mean H-scoring system according to Hirsch FR et al. [26] was also tested, but showed mostly a weak and non-significant prognostic and predictive value in this study. High intensity (strong) of the staining (membranes and nucleus) and a staining percentage > 5% (nucleus) were the two most valuable and efficient cut-off levels in the evaluation of this series. The cut-off level > 5%, indicating score = 0, has been use in the literature since 1995, when published by Sinicrope FA et al. [27].

### Statistical methods

In the statistical analyses the Pearson chi-square, the t-test, ANOVA statistics, logistic regression analysis, Cox proportional hazard regression analysis, Kaplan-Meier technique, and log-rank test for differences between proportions, mean values, median values, binary outcome, and survival curves. A *p*-value < 0.05 (double-sided) was regarded as statistically significant. The Statistica (version 12, 2013) software package (StatSoft, Inc., Tulsa, OK 74104 USA) was used in the statistical analyses.
